# Photosynthesis of Au/TiO_2_ nanoparticles for photocatalytic gold recovery from industrial gold-cyanide plating wastewater

**DOI:** 10.1038/s41598-022-24290-7

**Published:** 2022-12-19

**Authors:** Naphaphan Kunthakudee, Prakorn Ramakul, Karn Serivalsatit, Mali Hunsom

**Affiliations:** 1 Department of Chemical Engineering, Faculty of Engineering, Mahidol University, Phuttamonthon 4 Road, Salaya, Phuttamonthon, Nakhon Pathom, 73170 Thailand; 2 Department of Chemical Engineering, Faculty of Engineering and Industrial Technology, Silpakorn University, Nakhon Pathom, 73000 Thailand; 3 Department of Materials Science, Faculty of Science, Chulalongkorn University, Phayathai Road, Pathumwan, Bangkok, 10330 Thailand; 4 Associate Fellow of Royal Society of Thailand (AFRST), Bangkok, 10300 Thailand

**Keywords:** Environmental sciences, Engineering, Materials science, Nanoscience and technology

## Abstract

A series of Au_*x*_/TiO_2_ nanoparticles (NPs) with different gold loadings (*x* = 0.1–1.0 wt%) was synthesized by the photodeposition and then employed as photocatalysts to recover precious component from the industrial gold-cyanide plating wastewater. Effects of Au loading, catalyst dosage and types of hole scavenger on the photocatalytic gold recovery were investigated under ultraviolet–visible (UV–Vis) light irradiation at room temperature. It was found that different Au loadings tuned the light absorption capacity of the synthesized photocatalysts and enhanced the photocatalytic activity in comparison with the bare TiO_2_ NPs. The addition of CH_3_OH, C_2_H_5_OH, C_3_H_8_O, and Na_2_S_2_O_3_ as a hole scavenger significantly promoted the photocatalytic activity of the gold recovery, while the H_2_O_2_ did not. Among different hole scavengers employed in this work, the CH_3_OH exhibited the highest capability to promote the photocatalytic gold recovery. In summary, the Au_0.5_/TiO_2_ NPs exhibited the best photocatalytic activity to completely recover gold ions within 30 min at the catalyst dosage of 0.5 g/L, light intensity of 3.20 mW/cm^2^ in the presence of 20 vol% CH_3_OH as hole scavenger. The photocatalytic activity slightly decreased after the 5th cycle of recovery process, indicating its high reusability.

## Introduction

According to a fast development of communication and informative technology in our country together with the launch of 5G technology, and also the increasing demand of electronic devices, it is expected that the printed circuit board manufacturing industry will continuously grow during 2021–2023^1^. To prepare the surfaces of electrical contacts and the wire bonding pads of semiconductor devices, the gold plating is widely carried out due to the excellent properties of metallic gold including high electrical conductivity, high reliability, and high corrosion resistance^2,3^. Through the gold deposition is effectively formed on the nickel-phosphorous (Ni–P) coating deposited on copper (Cu) surface, the plating solution is generally unstable due to the presence of reductant. Thus, the potassium dicyanoaurate (K[Au(CN)]_2_) is usually used as the gold precursor because of its high stability and capability to form an excellent gold film^2,4–6^. Therefore, the wastewater generated from this process contains high content of gold-cyanide complexes as well as the chemicals used in the surface preparation process. Discharging of precious metal such as gold is an economic loss. Thus, this wastewater is conducted to recover gold by electrolysis and ion exchange process^6^. However, both processes are expensive and require precise control. Therefore, many processes have been developed and applied to recover gold from the gold-containing wastewater such as electrochemical process^7–10^, solvent extraction^11,12^, and adsorption^13–15^.

Another promising process that can be used to recover gold from the gold-cyanide plating wastewater is the photocatalytic process because of its environmentally friendly, ease of operation and control and able to operate at ambient condition with low operating cost^16,17^. This process does not require the external supplied electricity and electrodes like an electrochemical process, not require specific extractant like a solvent extraction and not require high surface containing adsorbents like the adsorption. This process can be taken place in the presence of irradiated light, that has the photon energy equal or greater than its band gap energy (*E*_*g*_)^18,19^. Then, an electron (e^−^) is excited from the valence band (VB) to the conduction band (CB), leaving a positive hole (h^+^) (reaction (R1)). In the presence of gold-cyanide complexes, the deposition of metallic gold on the photocatalyst surface will occur simultaneously with the release of free cyanide ions ^6^ as expressed by reactions (R2) and (R3). The gold recovery rate from the [Au(CN)_2_]^−^ is generally sluggish due to its low reduction potential (− 0.57 V/NHE)^6^. Therefore, many strategies have been carried out to recover gold from the gold-cyanide containing solution via the use of different photocatalysts as well as different operating conditions.

R1 $${\text{Semiconductor}} \to {\text{h}}^{ + } + {\text{ e}}^{ - }$$

R2 $$\left[ {{\text{Au}}\left( {{\text{CN}}} \right)_{{2}} } \right]^{ - } + {\text{ e}}^{ - } \to {\text{Au}} + {\text{2CN}}^{ - }$$

R3 $${\text{2CN}}^{ - } + {\text{ 4OH}}^{ - } + {\text{ 4h}}^{ + } \to {\text{2OCN}}^{ - } + {\text{ 2H}}_{{2}} {\text{O}}$$

Recently, it was reported that the TiO_2_/GrSiO_2_ exhibited the photocatalytic activity to remove gold ions from gold-cyanide plating solutions higher than the bare TiO_2_^6^. The release of free cyanide ions from the stable metal cyanocomplexes can be achieved by an increase in the availability of cyanide for the subsequent oxidation treatment. The presence of hole scavenger such as methanol (CH_3_OH) can promote the deposition of metallic Au NPs on the surface of utilized photocatalysts. The use of ZnS can induce the reduction of the gold-cyanide complexes as well as the reverse oxidation of gold NPs via the photogenerated holes at VB^20^. Approximately 38% of gold was recovered within 120 min via the use of Na_2_SO_3_ as the hole scavenger. The ZnO photocatalyst exhibited a high selectivity to recover gold ions from the potassium gold-cyanide wastewater due to its appropriate VB position^21^. The crystalline quality of the ZnO nanopowder affected positively the efficiency of gold recovery. A complete gold recovery can be achieved with 30 min in the presence of 10 vol% CH_3_OH. The interaction between the rGO and TiO_2_ in the rGO/TiO_2_ composite can alleviate the rate of electron–hole (e^−^–h^+^) recombination and enhance the charge transfer between RGO and TiO_2_ structure, thus promoting the photocatalytic gold recovery from the gold-cyanide complex solution ^22^. Based on mentioned above, it can be noted the photocatalytic activity of gold recovery depeneds upon various parameters, particularly the photocatalyst type, band position and hole scavenger.

In this work, a series of Au_*x*_/TiO_2_ was synthesized via the photodeposition using the commercial TiO_2_ as based material. Effects of gold loading on morphology and optical property of the obatined Au_*x*_/TiO_2_ NPs as well as the photocatalytic acitivity for gold recovery from the industrial plating wastwater were explored. The optimum hole scavenger type and catalyst dosage were also determined.

## Methods

### Property of utilized gold-cyanide plating wastewater

The utilized gold-cyanide plating wastewater was generously offered from the circuit board industry located in Phra Nakhon Si Ayutthaya, Thailand. This solution has a light-green clear color with the pH of 8.4–9.2. It contains high concentration of Au ions (10–15 mg/L) and a trace quantity of Cu, Ni, potassium (K), zinc (Zn) of less than 0.06, 0.46, 202.85 and 2.17 mg/L, respectively.

### Photocatalyst preparation and characterization

Gold nanoparticles at different contents (0.1–1.0 wt%) were deposited on the surface of commercial TiO_2_ (99.5%, Sigma-Aldrich) by the photodeposition using the chloroauric acid (99.9% HAuCl_4_·3H_2_O; Sigma-Aldrich) as the chemical precursor. Briefly, approximately 1.2 g of TiO_2_ (99.5%, Sigma-Aldrich) were dispersed in the 300 mL of 5 mg/L HAuCl_4_ solution in a double wall cylindrical glass reactor. The pH of the HAuCl_4_ solution was adjusted to 10 by 0.5 M sodium hydroxide (NaOH; Sigma-Aldrich). The glass reactor was then put on the hotplate stirrer (MSH-300, Biosan) and placed centrally in the UV-protected box. The external cold water was supplied to the jacket of the glass reactor and circulated for the whole experimental time to control the operating temperature (~ 30 °C). A solid–liquid mixture was constantly stirred at the rate of 400 rpm in the absence of light for 30 min to allow a good adsorption of gold ions on the surface of TiO_2_ NPs. Afterward, a high-pressure mercury lamp (400 W; RUV 533 BC, Holland) was turned on to generate the UV–visible light at the wavelength of 100–600 nm. The incident light intensity at the reactor was kept constantly at around 3.20 mW/cm^2^. When a complete photodeposition was achieved (~ 1 h), the solid NPs was first separated from a solid–liquid mixture by centrifugal at 11,000 rpm (Eppendorf, 5804R) and washed thoroughly with deionized (DI) water. The ready-to-use Au_0.1_/TiO_2_ NPs was obtained after drying at 80 °C for 3 h. The similar procedure was carried out to prepare Au_0.3_/TiO_2_, Au_0.5_/TiO_2_ and Au_1.0_/TiO_2_ via the use of HAuCl_4_ solution at gold ion concentrations of 15, 25 and 50 mg/L, respectively.

The morphology and optical property of all synthesized Au_*x*_/TiO_2_ NPs as well as the TiO_2_ were characterized as following. XRD patterns were taken on a Bruker D2 Phaser diffractometer using Cu Kα X-ray). The anatase fraction and average crystallite size of all photocatalyst NPs were computed via Spurr and Myers equation (Eq. 1) and Debye–Scherrer equation (Eq. 2), respectively^23^.

1 $$x_{A} = \frac{{100}}{{1 + \left[ {I_{R} /\left( {0.8I_{A} } \right)} \right]}}$$

where *x*_*A*_ is the anatase weight fraction, *I*_*A*_ is the relative reflection intensities of anatase and *I*_*R*_ is the relative reflection intensities of rutile.

2 $$D = \frac{{0.9\lambda }}{{\beta \cos \theta }}$$

where *D* is the average crystallite size (nm), *λ* is the wavelength of the X-ray radiation (0.154178 nm), *β* is the full width at half maximum intensity of the peak and *θ* is the diffraction angle.

Scanning electron microscopy (SEM) was carried out via a JSM-IT500HR and equipped with a JED-2300 energy dispersive X-ray (EDX) spectrometer at landing voltage of 15.0 kV and magnification × 3000. The content of metallic Au NPs on TiO_2_ was examined by inductively coupled plasma mass spectrometry (ICP-MS, PerkinElmer, NexION 2000) using hydrochloric acid (37% HCl, Merck) and nitric acid (68% HNO_3_, BDH) at volume ratio of 3:1 as extractant. High-resolution transmission electron microscopy (HRTEM) was taken via a JEM-3100F with an accelerating voltage of 300 kV. XPS data were harvested from Axis Supra^+^ (Kratos, UK) with a Delay Line detector (DLD) and a monochromatic Al Kα (*hν* = 1,486.6 eV) source. The spectra were calibrated with the C1s peak of adventitious carbon at binding energy of 284.8 eV to minimize the error of binding energies within the range of ± 0.1 eV. The UPS mode of this analysis allowed to estimate the value of Fermi edge energy (*E*_*f*_) and cutoff edge energy (*E*_*c*_), which can be used to compute the photocatalytic work function (Φ) as well as the valence band energy (*E*_*v*_) and conduction band energy (*E*_*c*_) according to Eqs. (3)–(5)^24–26^.

3 $$\Phi^{{}} =^{{}} hv - \left( {E_{f} - E_{c} } \right)$$

4 $$E_{V}^{{}} =^{{}} \Phi - E_{a}$$

5 $$E_{C}^{{}} =^{{}} \Phi - E_{g} - E_{a}$$

where Φ is the work function of photocatalysts, *hv* is the excitation source which equals to 21.2 eV for helium (He), *E*_*f*_ is the Fermi edge energy, *E*_*c*_ is the cutoff edge energy, *E*_*V*_ is the valence band energy, *E*_*C*_ is the conduction band energy, *E*_*a*_ is the electron affinity of TiO_2_ NPs (3.9 eV^27^) and *E*_*g*_ is the band gap energy obtained from UV–Vis analysis and Tauc’s plot.

N_2_ adsorption/desorption isotherms were detected using a Multipoint Surface Area Analyzer (Tristar II3020, Micromeritics) via the Brunauer–Emmett–Teller (BET) method. The photoluminescence (PL) data were taken on a Perkin-Elmer LS-55 Luminescence Spectrometer in air at ambient temperature with a 290 nm cut-off filter. The PL signals were collected in the range of 350–550 nm using a standard photomultiplier. UV–Vis absorption spectra were monitored over the wavelength range of 200−800 nm via a Cary300 UV–Vis spectrophotometer (Agilent).

### Photocatalytic activity of Au_*x*_/TiO_2_ for gold recovery

The experimental set up for testing the photocatalytic activity of the synthesized Au_*x*_/TiO_2_ NPs for gold recovery from gold-cyanide plating wastewater was similar to the previous section. That is, approximately 0.6 g of respective Au_*x*_/TiO_2_ was dispersed in 300 mL of gold-cyanide plating wastewater in the presence of selected hole scavengers including hydrogen peroxide (H_2_O_2_, BDH Laboratory), sodium thiosulphate (Na_2_S_2_O_3_, KemAus), methanol (CH_3_OH, QReC), ethanol (C_2_H_5_OH, QReC), *n*-propanol (*n*-C_3_H_8_O, KemAus), *i*-propanol (*i*-C_3_H_8_O, QReC) and glycerol (C_3_H_8_O_3_, KemAus). The solid–liquid mixture was stirred constantly at 300 rpm in dark environment for 30 min to allow a thoroughly dispersion and adsorption of gold complex species on the photocatalyst surface. Then, the system was irradiated by UV–Vis light (100–600 nm) generated by a high-pressure mercury lamp (400 W; RUV 533 BC, Holland). The light intensity was fixed over the whole experimental period at 3.20 mW/cm^2^. The temperature of the reactor was controlled at 28–30 °C by the water circulation at the reactor jacket using the circulating pump (PMD-0311, Sanso, Japan). As the reaction proceeded, approximately 5 mL of solution was taken at particular time and subjected to centrifuge at 11,000 rpm (5804R, Eppendorf) to separate the solid NPs from solution. The remining concentration of gold ions in solution at particular time was measured by flame atomic absorption spectrometry (Flame-AAS, Analyst 200 + flas 400; Perkin-Elmer). The reduction rate of gold ions from the gold-cyanide plating wastewater was fitted via the Langmuir–Hinshelwood model according to Eq. (6)^28^.

6 $$C/C_{{0^{{}} }} =^{{}} \exp \left( { - kt} \right)$$

where *C*_0_ is the initial gold ion concentration (mg/L), *C* is the gold ion concentration at particular time (mg/L), *t* is the reaction time (min) and *k* is the first-order reaction rate constant (min^−1^).

## Results and discussion

### Morphology and optical property of synthesized Au_x_/TiO_*2*_

**Figure 1:**
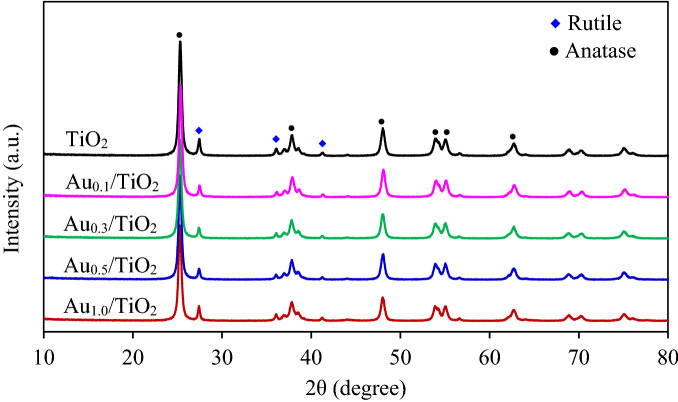
Representative XRD spectra of commercial TiO_2_ and Au_*x*_/TiO_2_ NPs synthesized by photodeposition at different Au loadings in the range of 0.1–1.0 wt%.

To shed some light on the crystallite structure of all Au_*x*_/TiO_2_ photocatalysts, the XRD analysis was first carried out. As depicted in Fig. 1, the XRD pattern of the original TiO_2_ NPs exhibited the characteristic peaks of anatase at 2θ of 25.3°, 37.8°, 48.0°, 53.9°, 55.1° and 62.7°, relating to the crystal plane of (101), (004), (200), (105), (221) and (204), respectively (JCPDS file no. 21-1272). Besides, it showed the principal characteristic peaks of rutile phase at 2θ of 27.4°, 36.0°, and 41.2°, relating to the crystal plane of (110), (101), and (111), respectively, (JCPDS file no. 21-1276). No characteristic peaks of Au NPs were observed, probably due to the low content and the high dispersion of Au. The anatase/rutile (A/R) ratio of all Au_*x*_/TiO_2_ NPs estimated from the Spurr and Myers equation (Eq. 1) were around 0.83–0.87. Also, the average crystallite size of TiO_2_ in anatase (A(101)) and rutile (R(110)) phases computed from Debye–Scherrer equation (Eq. 2) were around 21 and 30 nm, respectively. Both A/R ratio and crystallite size of TiO_2_ deviated very slightly from the original TiO_2_ NPs, suggesting that the deposition of Au NPs via the utilized procedure did not change the main characteristic of the parental TiO_2_ NPs.

**Figure 2:**
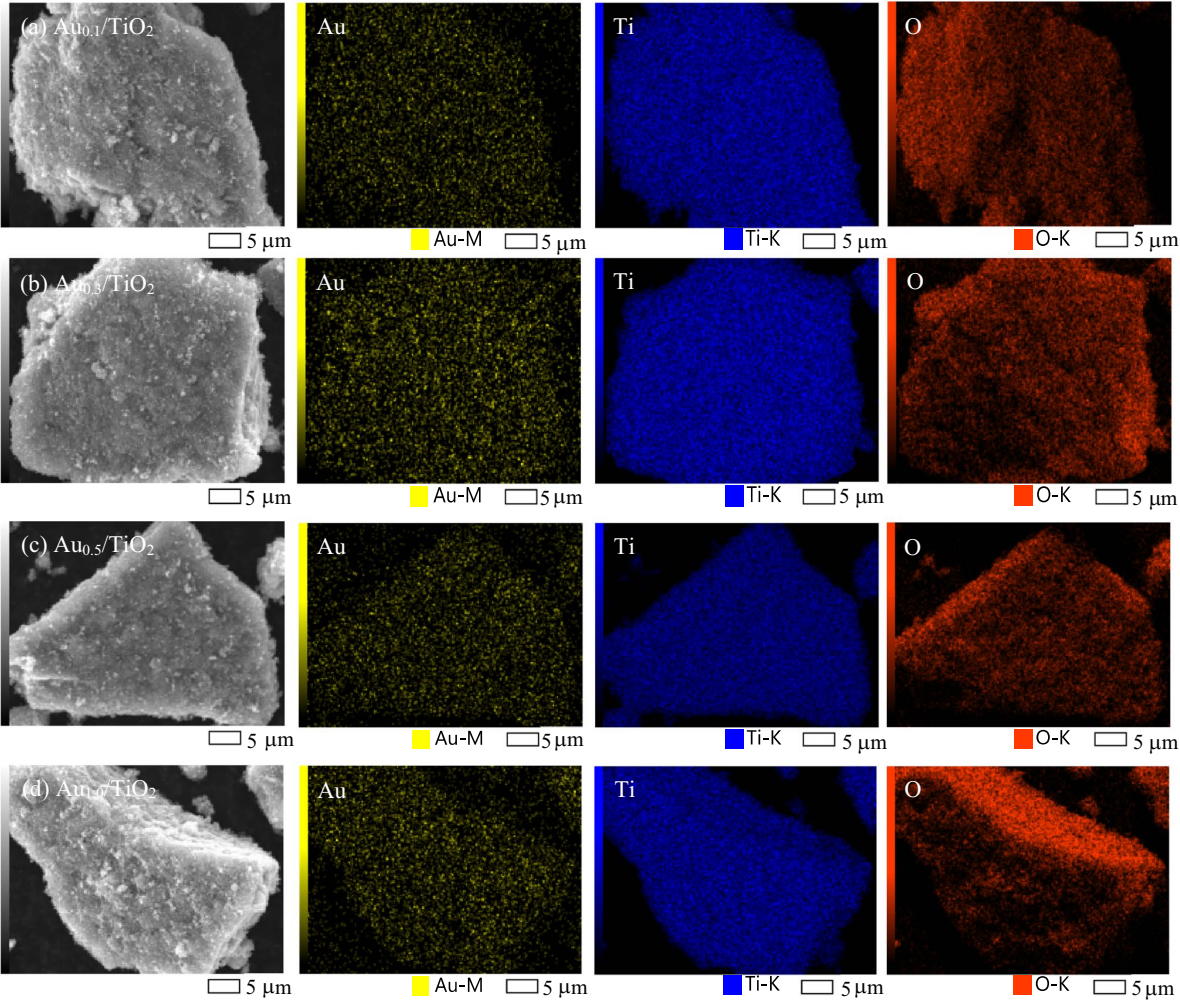
Representative SEM images and EDX element mapping at 3000 × magnification of Au_*x*_/TiO_2_ NPs synthesized by photodeposition at different gold loadings in the range of 0.1–1.0 wt%.

**Figure 3:**
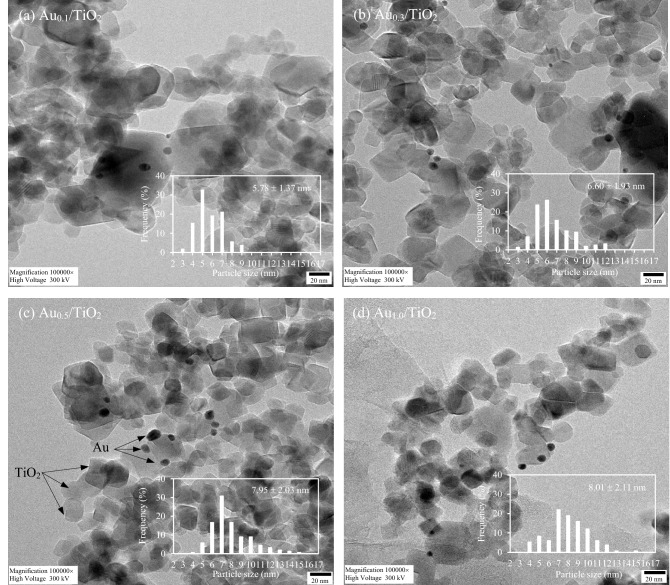
Representative HRTEM of Au_*x*_/TiO_2_ NPs synthesized by photodeposition and their particle size distributions at different gold loadings in the range of 0.1–1.0 wt%.

The presence of deposited Au NPs on the surface TiO_2_ was then explored via the SEM–EDX analysis. As displayed in Fig. 2, a uniform distribution of Au NPs was observed for all loadings. The amount of deposited gold examined by both SEM–EDX and ICP was closed to the preset content as summarized in Table 1. The average particle size of deposited Au NPs was then examined using a high-resolution TEM analysis. As clearly shown in Fig. 3, all HRTEM images showed a clear feature of both TiO_2_ and Au NPs. The TiO_2_ NPs exhibited a spherical shape of anatase crystallites (~ 21 nm) and also an angular shape of rutile crystallites (~ 30 nm). The Au NPs exhibited a pseudo-spherical shape with average particle size in the range of 5.8–8.0 nm (Table 1). According to the particle size distribution, it seems to be that the size of Au NPs was largely dependent on the Au loading at low values. That is, the size of Au NPs increased from 5.78 to 7.95 nm as the increase of gold loadings from 0.1 to 0.5 wt% and slightly increased from 7.95 to 8.01 nm as the increase of gold loadings from 0.5 to 1.0 wt%. The size variation of Au NPs on the surface of TiO_2_ might responsible for the optical property as well as the photocatalytic activity of the Au_*x*_/TiO_2_ photocatalysts.

**Table 1:** Morphology and optical property of all synthesized Au_*x*_/TiO_2_ and the parental TiO_2_ NPs.

Catalysts	A/R ratio^a^	Crystallite size^b^ (nm)		Au content (wt%)		Size of Au NPs^c^ (nm)	Textural property		*E*_*g*_ (eV)	*E*_*c*_ (eV)	*E*_*f*_ (eV)
		TiO_2_ (A)	TiO_2_ (R)	SEM–EDX	ICP		BET area (m^2^/g)	Total pore volume (cm^3^/g)			
TiO_2_	0.839	21.26	30.51	–	–		54.4	0.1525	3.35	3.38	18.20
Au_0.1_/TiO_2_	0.880	21.43	30.29	0.12	0.14	5.78 ± 1.37	53.3	0.2929	3.24	3.23	18.20
Au_0.3_/TiO_2_	0.875	21.43	30.29	0.35	0.35	6.60 ± 1.93	53.2	0.2825	3.25	3.40	18.40
Au_0.5_/TiO_2_	0.879	21.43	30.86	0.56	0.56	7.95 ± 2.03	53.2	0.2760	3.22	3.34	18.40
Au_1.0_/TiO_2_	0.838	21.43	30.29	1.17	1.15	8.01 ± 2.11	55.7	0.4043	3.26	3.25	18.20

^a^Estimated from XRD analysis using Spurr and Myers equatio

^b^Estimated from XRD analysis using Debye–Scherrer equation.

^c^ Estimated from HRTEM analysis.

**Figure 4:**
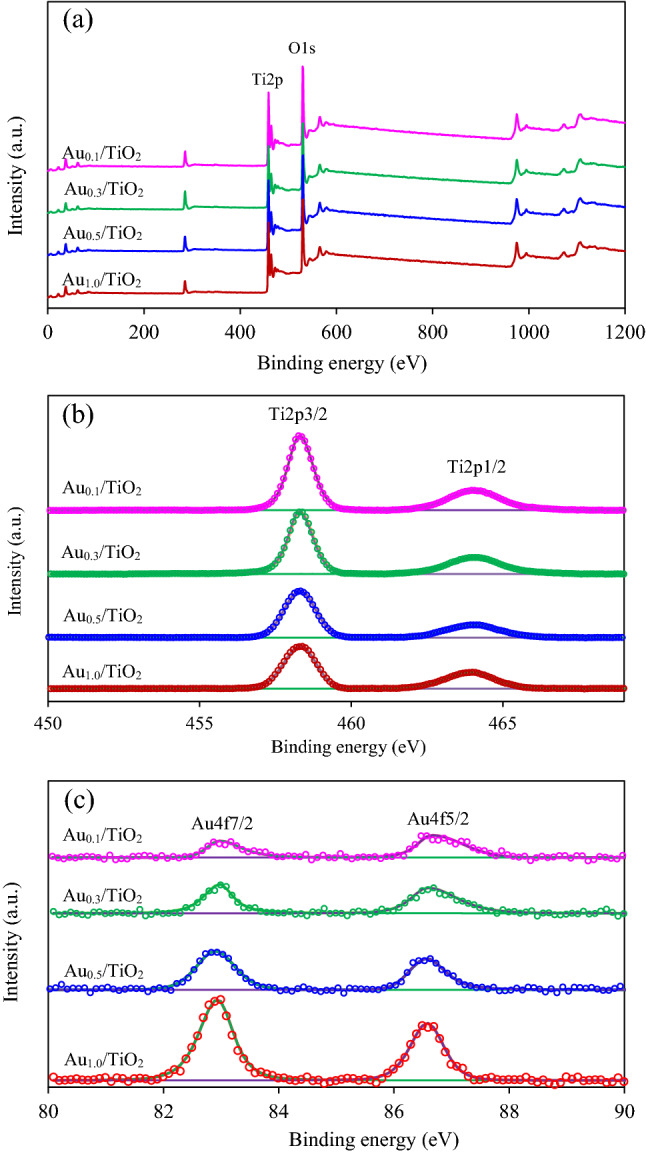
(**a**) Representative wide scan XPS spectra, (**b**) Ti2p and (**c**) Au 4f XPS spectra of Au_*x*_/TiO_2_ NPs photocatalysts synthesized by photodeposition and their particle size distributions at different gold loadings in the range of 0.1–1.0 wt%.

To probe the presence of chemical elements as well as the oxidation state of gold on the surface of TiO_2_, the XPS analysis was carried out. As demonstrated in Fig. 4a, the survey XPS showed the intensive peaks of Ti2p and O1s of Ti and O species at binding energy of around 458.3 and 530.2 eV, respectively. The high resolution XPS spectra of Ti2p components in the parental TiO_2_ structure exhibited two symmetric spectra at binding energy of 458.3 and 464.1 eV (Fig. 4b), corresponding to the spin-orbital doublet of the Ti2p3/2 and Ti2p1/2 components^29,30^, which indicates the presence of Ti^4^^+^ species in TiO_2_ NPs^31^. The Ti2p XPS spectra of Au_*x*_/TiO_2_ NPs also revealed the symmetric spectra of Ti2p3/2 and Ti2p1/2 components without any shifting of binding energy compared with those of parental TiO_2_ NPs. This suggested that the addition of Au NPs on the surface of TiO_2_ by the photodeposition cannot enhance the formation of the disorder and/or defective structure of TiO_2_. The Au4f XPS spectra of all Au_*x*_/TiO_2_ NPs showed the core-level electrons at binding energy of ~ 82.9 and ~ 86.5 eV, respectively (Fig. 4c), assigning to the Au4f7/2 and Au4f5/2 of metallic gold ^32,33^. The intensity of core-level features increased linearity as the increasing gold loading. The Au4f spectra of gold ions were not detected, suggesting that the original gold ions in gold chloride precursor were completely reduced to metallic gold via the photodeposition.

**Figure 5:**
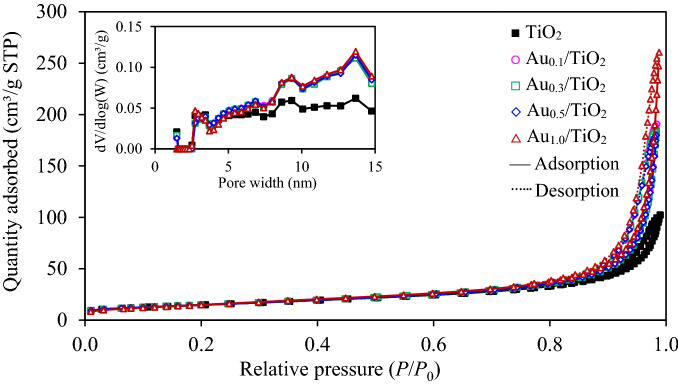
Representative N_2_ adsorption/desorption isotherms of commercial TiO_2_ and Au_*x*_/TiO_2_ NPs synthesized by photodeposition at different gold loadings in the range of 0.1–1.0 wt%.

Regardless the textural property of all synthesized samples, the N_2_ adsorption/desorption isotherms of all synthesized Au_*x*_/TiO_2_ NPs together with the parental TiO_2_ NPs were examined and displayed in Fig. 5. All synthesized samples exhibited the Type IV adsorption/desorption isotherm according to the classification of the International Union of Pure and Applied Chemistry (IUPAC). The presence of the H4-hysteresis loop was also observed for all samples, indicating the formation of the slit-like porous structures^34^. The variation of adsorbed quantity as a function of pore width during 1.48–14.76 nm was also shown as the inset of Fig. 5. Within this pore width range, all Au_*x*_/TiO_2_ NPs exhibited a larger adsorbed quantity than the parental TiO_2_ NPs. Quantitatively, the BET surface area and total pore volume of all Au_*x*_/TiO_2_ were summarized in Table 1. The Au_1.0_/TiO_2_ exhibited the BET surface area slightly higher than that of other Au_*x*_/TiO_2_ NPs as well as the parental TiO_2_, probably due to its well dispersion on the surface of TiO_2_^26,35^, which can enhance a high adsorption capacity.

**Figure 6:**
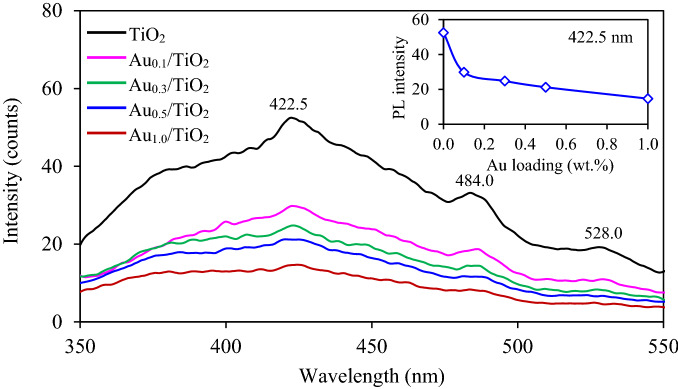
Representative photoluminescence spectra (PL) of commercial TiO_2_ and Au_*x*_/TiO_2_ NPs synthesized by photodeposition at different gold loadings in the range of 0.1–1.0 wt.%.

The qualitative recombination rate of e^−^–h^+^ pairs was then determined using the photoluminescence (PL) spectrometer. An intense peak of PL signal indicates a high recombination rate of e^−^–h^+^ pair. From the plot, the TiO_2_ NPs revealed three highly broad PL emission peaks, centered at 422.5, 484.0 and 528.0 nm (Fig. 6), indicating a fast e^−^–h^+^ recombination. A high-energy spectrum at 422.5 nm is associated from the band edge excitation of TiO_2_, other spectra are attributed to the electron transition by the state of oxygen vacancies and/or defective structure^36,37^. All Au_*x*_/TiO_2_ NPs showed lower intense PL spectra than those of TiO_2_ NPs, indicating that the presence of Au NPs can suppress the rate of e^−^–h^+^ recombination. Among all Au_*x*_/TiO_2_ NPs, the intensity of PL spectra decreased as the increase of gold loading (inset of Fig. 6). This can be explained in terms of the charge separation at Schottky junction and the localized surface plasmon resonance (LSPR) phenomenon of Au NPs^38^. High deposited gold may function as the electron sink, allowing the migration and movement of electrons from the surface to the bulk which consequently promote the e^−^–h^+^ separation as well as suppress the e^−^–h^+^ recombination^39^.

**Figure 7:**
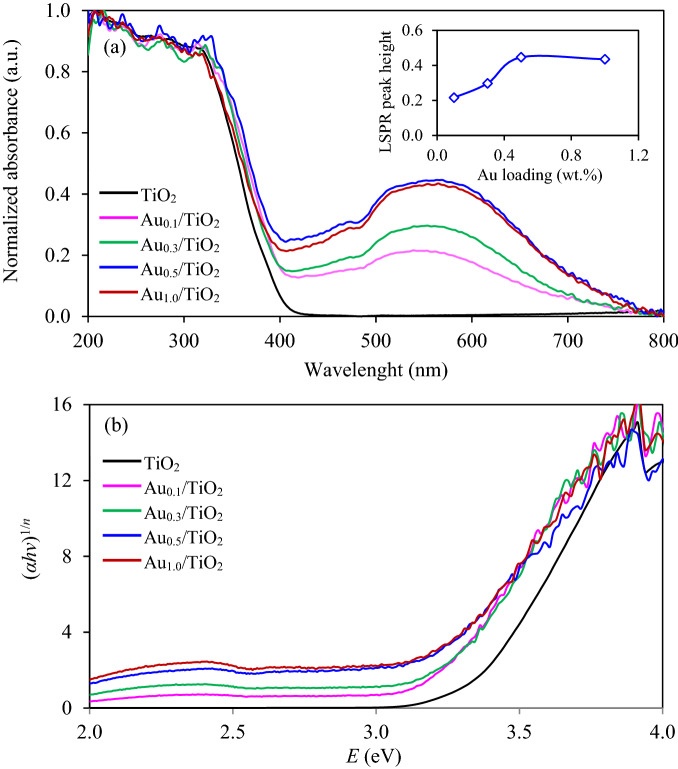
(**a**) UV–Vis absorption spectra and (**b**) Tauc’s plots of commercial TiO_2_ and Au_*x*_/TiO_2_ NPs synthesized by photodeposition at different gold loadings in the range of 0.1–1.0 wt%.

To further explore the optical property of all synthesized samples, the light absorption capacity of all synthesized Au_*x*_/TiO_2_ NPs as well as the parental TiO_2_ was then analyzed. As illustrated in Fig. 7a, the parental TiO_2_ NPs exhibited a strong absorbance during the ultraviolet (UV) light regime (*λ* < 400 nm) due to its visible light inertness in nature. All Au_*x*_/TiO_2_ NPs exhibited strong absorption spectrum in the visible light region (*λ* > 400 nm) with the broad absorption features ~ 540–570 nm, assigning to the LSPR behavior of deposited Au NPs^33^. The intensity of LSPR peaks increased as the increase of nominal gold loading up to 0.5 wt% and dropped slightly afterward (inset of Fig. 7a). A high light absorption might enhance the harvesting of incident light and also promote the photocatalytic activity. Slight red-shift in the plasmon position was observed with the increased nominal gold loading due to the size increase of Au NPs^40^. Tauc plots or plots of (*αhν*)^1/*n*^ versus photon energy (*E*) computed from the UV–Vis absorption spectra (Fig. 7a) are shown in Fig. 7b. The *E*_*g*_ of each sample can be obtained by extrapolation the linear portion of this plot to intercept the *x*-axis. As summarized in Table 1, the *E*_*g*_ energy of TiO_2_ decreased importantly from 3.35 to 3.22 eV with addition of Au NPs.

**Figure 8:**
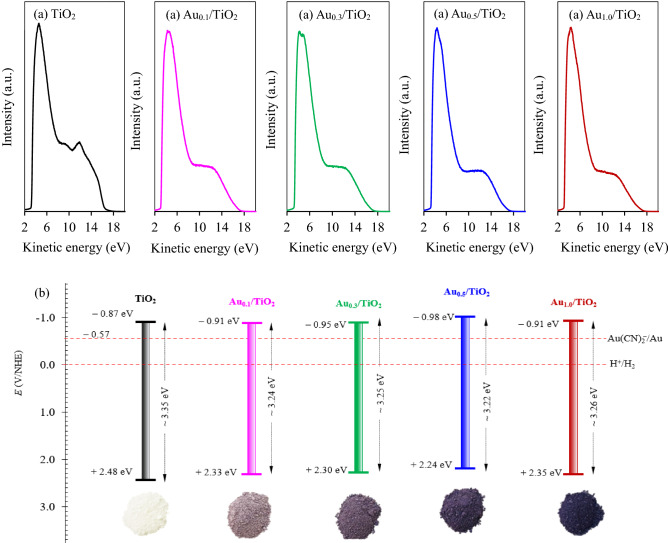
(**a**) Intensity-kinetic energy plots of commercial TiO_2_ and Au_*x*_/TiO_2_ NPs synthesized by photodeposition at different gold loadings in the range of 0.1–1.0 wt% and (**b**) sketch of their band position.

Figure 8a illustrates the intensity-kinetic energy plots of commercial TiO_2_ and all synthesized Au_*x*_/TiO_2_ NPs. Values of *E*_*f*_ and *E*_*c*_ taken respectively from the *x*-axis interception at high- and low binding energies of this plot are summarized in Table 1. Both obtained energy values were then used to compute the Φ as well as the *E*_*V*_ and *E*_*C*_ according to Eqs. (3)–(5). It was obtained that the Φ of TiO_2_, Au_0.1_/TiO_2_, Au_0.3_/TiO_2_, Au_0.5_/TiO_2_ and Au_1.0_/TiO_2_ NPs could be determined to be 6.38, 6.23, 6.20, 6.14 and 6.25 eV, respectively. Figure 8b represents the briefly sketch of band position of all synthesized Au_*x*_/TiO_2_ and commercial TiO_2_ NPs. It is noteworthy that both synthesized Au_*x*_/TiO_2_ and the parental TiO_2_ NPs had a more negative conduction band level than the reduction potential of [Au(CN)_2_]^−^, indicating their ability to act as the photocatalyst for gold reduction from the gold-cyanide plating wastewater.

### Photocatalytic activity of Au_*x*_/TiO_2_ for gold recovery

#### Effect of Au loading on TiO_2_ NPs

**Figure 9:**
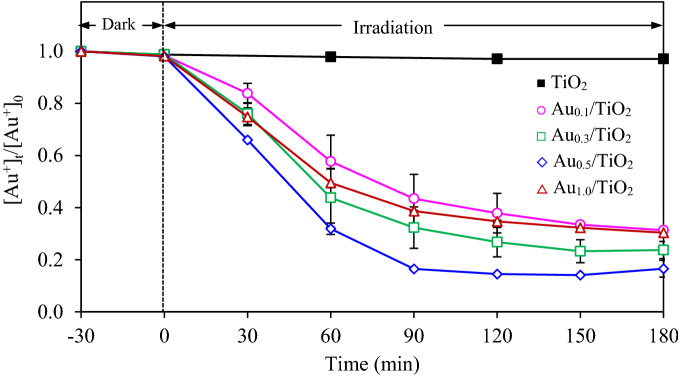
Variation of gold recovery via different Au_*x*_/TiO_2_ photocatalysts at dosage of 2 g/L and light intensity of 3.20 mW/cm^2^ in the absence of hole scavenger.

The effect of gold content (0.1–1.0 wt%) deposited on the surface of TiO_2_ was first explored for the photocatalytic gold recovery from the gold-cyanide plating wastewater at the photocatalyst dosage of 2 g/L and light intensity of 3.20 mW/cm^2^ in the absence of hole scavenger. As shown in Fig. 9, the concentrations of gold ions did not change at particular time in the presence of TiO_2_ NPs, while it decreased significantly in the presence of Au_*x*_/TiO_2_ NPs. Among all Au_*x*_/TiO_2_ samples, the Au_0.5_/TiO_2_ NPs can achieve a high reduction of gold ions from the plating wastewater. This indicated that the Au_0.5_/TiO_2_ exhibited the highest photocatalytic activity for gold recovery from the plating wastewater. Its apparent rate constant estimated from the Langmuir–Hinshelwood model (Eq. 6) was found to be at 0.0190 min^−1^, which was higher than those of TiO_2_, Au_0.1_/TiO_2_, Au_0.3_/TiO_2_, and Au_1.0_/TiO_2_ for 38.8, 2.1, 1.4 and 1.6-fold, respectively.

Taking into account the morphology and optical property of all photocatalysts, it can be seen that the variation trend of the photocatalytic activity of all photocatalysts did not correlate with the content or size of deposited gold NPs, height of PL spectra as well as the total pore volume (Table 1). However, it slightly changed corresponding to the variation of the *E*_*g*_ and height of LSPR peak. That is, the Au_0.5_/TiO_2_ NPs exhibited the lowest *E*_*g*_ of 3.22 eV and the highest height of LSPR peak and also depicted the highest photocatalytic gold recovery from the gold-cyanide plating wastewater. Interestingly, although the Au_1.0_/TiO_2_ NPs showed the lowest PL spectra, comparable *E*_*g*_ to Au_0.3_/TiO_2_ and comparable LSPR peak height to Au_0.5_/TiO_2_, it showed lower the photocatalytic activity for gold recovery than both Au_0.3_/TiO_2_ and Au_0.5_/TiO_2_ NPs. This might be due to its high deposited gold content that allowed a freely transfer of excited electron along the bulk phase of gold structure and thus in turn slowdown the photocatalytic activity due to their function as an electron sink^39^. Another possible reason might be due to the synergetic effect of electron transfer between the anatase, rutile and metallic gold^33^. That is, a high gold content can randomly deposit on the anatase–rutile interface and also on the isolated anatase or rutile crystallite. The former gave the positive effect to the photocatalytic of Au_*x*_/TiO_2_ NPs, while the latter gave the negative effect^33^. A high gold content probably induced a high proportion of Au/rutile, which consequently suppressed the photocatalytic activity of Au_*x*_/TiO_2_ NPs. Besides, a high gold loading might form the shadowing behavior, thus preventing the absorption of incident light of the photocatalyst NPs^33,41^.

#### Effect of hole scavengers

**Figure 10:**
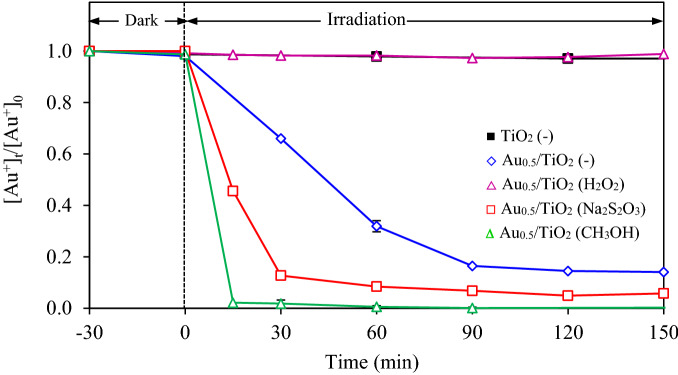
Variation of gold recovery via Au_0.5_/TiO_2_ photocatalyst at dosage of 2 g/L and light intensity of 3.20 mW/cm^2^ in the presence of different hole scavengers.

Since the photocatalytic recovery of gold from gold-cyanide plating wastewater is impelled by the photogenerated e^−^, a rapid recombination of photogenerated h^+^ and e^−^ may suppress the photocatalytic activity. To extend the lifetime of e^−^–h^+^ pairs, the removal of photogenerated h^+^ from the photocatalyst was carried out by introduction of sacrificial hole scavengers to the plating wastewater. In this part, three types of hole scavengers including H_2_O_2_, Na_2_S_2_O_3_ and CH_3_OH were first employed at identical quantity of 20 vol%. The variations of gold recovery via Au_0.5_/TiO_2_ photocatalyst at dosage of 2 g/L and light intensity of 3.20 mW/cm^2^ in the presence of different hole scavengers were depicted in Fig. 10. The addition of H_2_O_2_ did not promote the photocatalytic gold recovery via the utilized photocatalyst, while the addition of Na_2_S_2_O_3_ and CH_3_OH encouraged the photocatalytic rate of gold recovery. The apparent rate constants of the system using Na_2_S_2_O_3_ and CH_3_OH were 0.0688 min^−1^ and 0.1332 min^−1^ which were higher than that in the absence of hole scavenger of 3.6- and 7.0-fold, respectively.

The different photocatalytic activity of each hole scavenger might be attributed to the formation of different ionic species when the hole scavengers reacted with the photogenerated hole^42^. That is, the active form of hole scavenger in the presence of H_2_O_2_ was the OH^−^, which is coming from the reaction of H_2_O_2_ via the incident light or the photogenerated e^−^ according to reactions (R4)–(R6)^43,44^. Based on these reactions, the use of H_2_O_2_ as the hole scavenger consumes the photogenerated e^−^ competitively with the photocatalytic reduction of gold ions to metallic Au NPs, which may suppress the photocatalytic gold recovery of Au_*x*_/TiO_2_ NPs. Besides, the lack of photocatalytic gold recovery in the presence of H_2_O_2_ might be originated from its fast decomposition to H_2_O and O_2_, which can be experimentally observed immediately after the addition of this hole scavenger. A low photocatalytic activity in the presence of H_2_O_2_ as the hole scavenger was also observed for the H_2_ production via the TiO_2_ nanotubes^45^.

R4 $${\text{H}}_{{2}} {\text{O}}_{{2}} + hv \to {\text{2HO}}^{\cdot}$$

R5 $${\text{H}}_{{2}} {\text{O}}_{{2}} + {\text{ e}}^{ - } \to {\text{HO}}^{\cdot} + {\text{ OH}}^{ - }$$

R6 $${\text{OH}}^{ - } + {\text{ h}}^{ + } \to {\text{HO}}^{\cdot}$$

Via the Na_2_S_2_O_3_, the thiosulphate ions ((S_2_O_3_)^2−^) can react with the photogenerated h^+^ at the VB position to form (S_4_O_6_)^2−^ according to reaction (R7)^46^. For comparison, when CH_3_OH reacted with the photogenerated holes in the Au_0.5_/TiO_2_ NPs, the methoxy radicals (CH_3_O·) may form according to reaction (R8)^37,42^. These generated radicals can effectively supply electrons to the catalyst surface due to its low standard reduction potential (reaction (R9))^47,48^, thus promoting the reduction of the adsorbed gold-cyanide species.

R7 $${2}\left( {{\text{S}}_{{2}} {\text{O}}_{{3}} } \right)^{{{2} - }} + {\text{ 2h}}^{ + } \to ({\text{S}}_{{4}} {\text{O}}_{{6}} )^{{{2} - }}$$

R8 $${\text{CH}}_{{3}} {\text{OH }} + {\text{ h}}^{ + } \to {\text{CH}}_{{3}} {\text{O}}^{\cdot} + {\text{ H}}^{ + } \left( {0.{\text{48 V}}/{\text{NHE}}} \right)$$

R9 $${\text{CH}}_{{3}} {\text{O}}^{\cdot} \to {\text{CH}}_{{2}} {\text{O }} + {\text{ e}}^{ - } + {\text{ H}}^{ + } \left( { - 0.{\text{95 V}}/{\text{NHE}}} \right)$$

**Figure 11:**
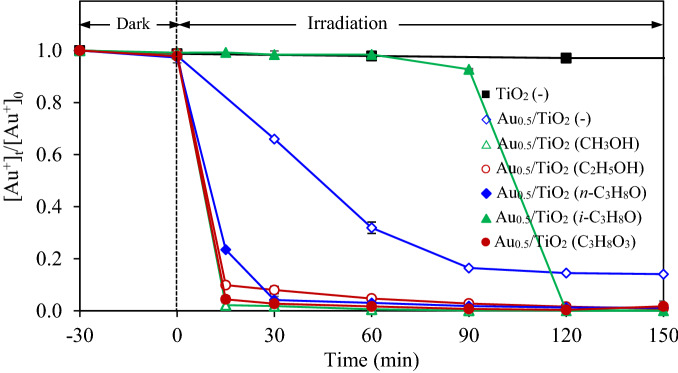
Variation of gold recovery via Au_0.5_/TiO_2_ photocatalyst at dosage of 2 g/L and light intensity of 3.20 mW/cm^2^ in the presence of different alcohols as the hole scavenger.

**Figure 12:**
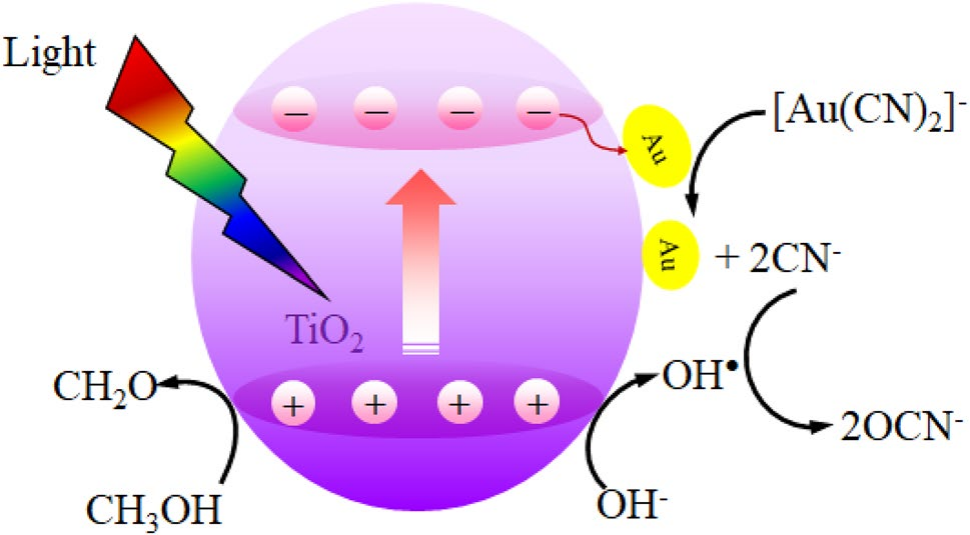
Possible mechanism of photocatalytic gold recovery from gold-cyanide containing solution over Au/TiO_2_ NPs in the presence of CH_3_OH as hole scavenger.

The effect of alcohol types including CH_3_OH, C_2_H_5_OH, *n*-C_3_H_8_O, *i*-C_3_H_8_O and C_3_H_8_O_3_ on the photocatalytic gold recovery via Au_0.5_/TiO_2_ photocatalyst was further explored at identical quantity of 20 vol% using the photocatalyst dosage of 2 g/L and light intensity of 3.20 mW/cm^2^. As shown in Fig. 11, the positive effect of hole scavenger on the photocatalytic gold recovery can be ranked as the order of CH_3_OH > C_3_H_8_O > C_2_H_5_OH > *n*-C_3_H_8_O > *i*-C_3_H_8_O. This is probably due to the effect of their different oxidation potentials. That is, the driving force of hole scavenging is dictated by the oxidation potential of the hole scavenger molecules^45^. The chemicals with low oxidation potential thermodynamically exhibits a rapid hole scavenging and vice versa^37,45^. Based on the obtained results, this fact is mostly conformed for the CH_3_OH (0.016 V/NHE), C_2_H_5_OH (0.084 V/NHE), *n*-C_3_H_8_O (0.100 V/NHE) and *i*-C_3_H_8_O (0.105 V/NHE)^37^, except the C_3_H_8_O (0.004 V/NHE). Although CH_3_OH has the oxidation potential four times higher than that of C_3_H_8_O_8_, it exhibited a higher positive effect on the photocatalytic gold recovery. This might be due to the electron dissemination of the generated CH_3_O^·^ radicals according to reaction (R9), which then reduce the adsorbed gold-cyanide species to metallic Au NPs. Interestingly, the system with *i*-C_3_H_8_O exhibited an extremely low gold reduction during the first 90 min although its oxidation potential is slightly lower than that of *n*-C_3_H_8_O. This might be attributed the effect of the molecular steric hindrance of this branched alcohol. That is, a high quantity of *i*-C_3_H_8_O at the early reaction period may obstruct each other to react with the photogenerated h^+^, thus lower the photocatalytic activity. However, as the time proceeded, part of this alcohol was consumed. Therefore, the photocatalytic activity increased due to the lessening of the steric hindrance effect. Based on the obtained results and literature^6,37,42,49^, the mechanism of photocatalytic gold recovery from the gold-cyanide plating wastewater on the Au/TiO_2_ NPs in the presence of CH_3_OH was roughly sketched as illustrated in Fig. 12.

#### Effect of catalyst dosage

**Figure 13:**
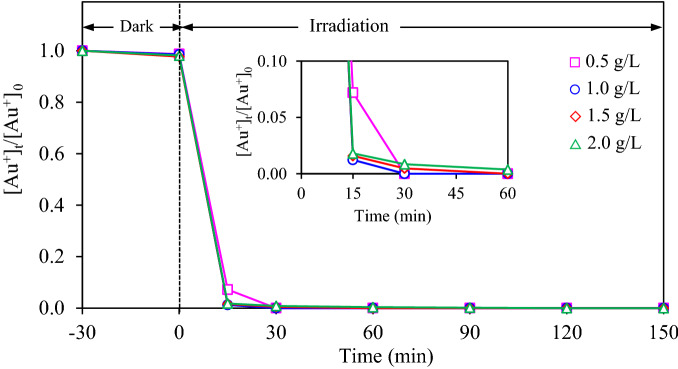
Variation of gold recovery via Au_0.5_/TiO_2_ photocatalyst at different dosages and light intensity of 3.20 mW/cm^2^ in the presence of 20 vol% CH_3_OH as the hole scavenger.

The effect of photocatalyst dosage (0.5–2.0 g/L) on the photocatalytic gold recovery was then examined at light intensity of 3.20 mW/cm^2^ in the presence of 20 vol% CH_3_OH. As displayed in Fig. 13, approximately 93% of gold ions was recovered within 15 min via the use of 0.5 g/L of Au_0.5_/TiO_2_ NPs, while greater than 98% was recovered via the same photocatalyst at the dosage of 1.0–2.0 g/L at the same time. A low photocatalytic activity to recover gold at low photocatalyst dosage might be due to the limitation of active site to proceed the photoreaction. Nevertheless, too high photocatalyst dosage also exhibited a low photocatalytic activity due to the light scattering effect as described by Beer-Lambert Law and also the light attenuation due to the self-shading behavior^50,51^. Thus, it can be noted that the optimum dosage of Au_0.5_/TiO_2_ NPs for the photocatalytic gold recovery from the spent gold-cyanide plating wastewater was 1.0 g/L.

Table 2 summarizes the comparative photocatalytic gold recovery from the gold-cyanide containing solution between this work and previous works. It is worth noting that the Au_0.5_/TiO_2_ photocatalyst synthesized in this work was on par with other previous works. It exhibited a higher photocatalytic activity than TiO_2_^52^, TiO_2_/GrSiO_2_^6^ and ZnS^20^ and comparable activity with ZnO^21^. With respect to rGO/TiO_2_, it is difficult to conclude that which photocatalyst type is better between rGO/TiO_2_ and Au_0.5_/TiO_2_ due to their high different initial gold ions concentration (~ 13.7-fold).

**Table 2:** Comparative photocatalytic gold recovery from the gold-cyanide plating wastewater via different photocatalysts under UV irradiation.

Photocatalyst	Operating condition				Recovery of Au NPs
	Matrix/initial Au ions concentration (mg/L)	Matrix/hole scavenger	Light source	Catalyst dosage (g/L)	
60%TiO_2_/GrSiO_2_^6^	[Au(CN)_2_]^−^/75 ppm	30 mM CH_3_OH	Medium pressure Hg lamp (150 W, 365 nm)	0.5	89% at 240 min
TiO_2_ (P25)^52^	[Au(CN)_2_]^−^/18 ppm	10 vol% CH_3_OH	Hg-Xe lamp (1000 W, ≥ 210 nm, 290 mW/cm^2^)	2.0	100% at 100 min
ZnS^20^	[Au(CN)_2_]^−^/40 ppm	10 mM Na_2_SO_3_	n.d. (254 nm)	~ 1.0	38% at 120 min
ZnO^21^	[Au(CN)_2_]^−^/60 ppm	10 vol% CH_3_OH	n.d.*	3.0	100% at 30 min
1.0%rGO/TiO_2_^22^	[Au(CN)_2_]^−^/205 ppm	10 mM *i*-C_3_H_8_O	LuzChem photoreactor (6-lamp, 365 nm)	1.0	30% at 180 min
Au_0.5_/TiO_2_ (This work)	[Au(CN)_2_]^−^/15 ppm	20 vol% CH_3_OH	High pressure Hg lamp (400 W, 365 nm, 3.20 mW/cm^2^	1.0	100% at 30 min

*Not detected.

**Figure 14:**
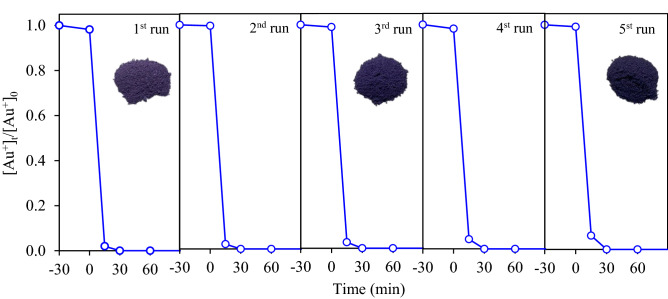
Repetitive gold recovery via Au_0.5_/TiO_2_ photocatalyst at loading of 1.0 g/L and light intensity of 3.20 mW/cm^2^ in the presence of 20 vol% CH_3_OH as the hole scavenger.

Figure 14 displays the variation of repetitive gold recovery from the gold-cyanide plating wastewater via the Au_0.5_/TiO_2_ photocatalyst at loading of 1.0 g/L and light intensity of 3.20 mW/cm^2^ in the presence of 20 vol% CH_3_OH as the hole scavenger. After each photocatalytic experiment, the utilized photocatalyst was washed delicately with DI water and dried in air at 80 °C for 3 h and then subjected to the next photocatalytic run. It can be seen that a fast decrease of gold ions was observed for all repetitive runs, indicating a high resuability of the synthesized Au/TiO_2_ NPs. The normalized concentration of remianing gold ions were around 0.02, 0.021, 0.027, 0.043 and 0.061 after the 1st, 2nd, 3rd, 4th and 5th run, respectively. The purple color of the fresh Au_0.5_/TiO_2_ NPs intensified with the increasing repetive runs, due to the increasing deposited Au NPs on photocatalyst surface^33,53^.

**Figure 15:**
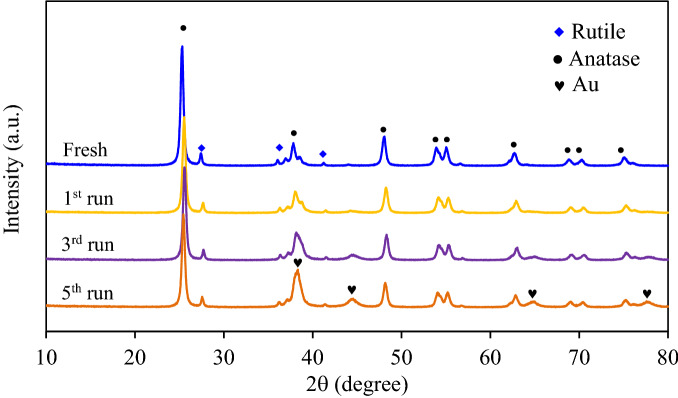
Representative XRD analysis of Au_0.5_/TiO_2_ photocatalyst after repetitive runs at loading of 1.0 g/L and light intensity of 3.20 mW/cm^2^ in the presence of 20 vol% CH_3_OH as the hole scavenger.

**Figure 16:**
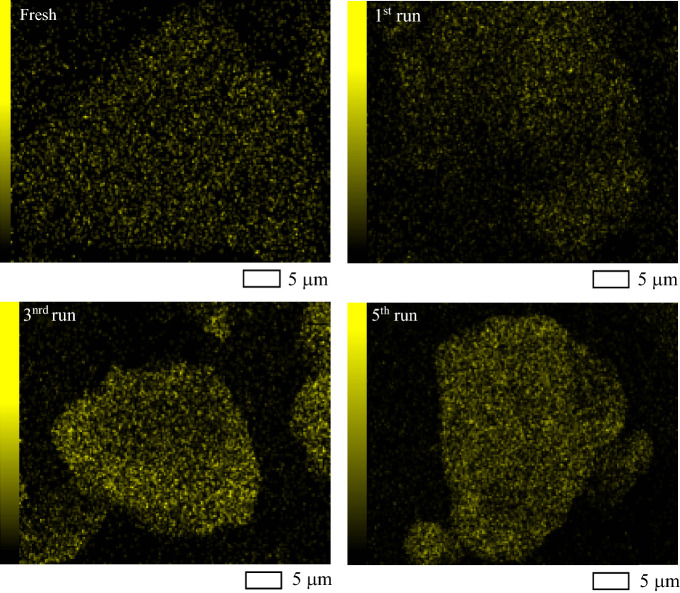
Representative Au mapping of Au_0.5_/TiO_2_ photocatalyst after repetitive runs at loading of 1.0 g/L and light intensity of 3.20 mW/cm^2^ in the presence of 20 vol% CH_3_OH as the hole scavenger.

The crystallite structure of Au_0.5_/TiO_2_ NPs after all repetitive experiments was also analyzed as shown in Fig. 15. The XRD pattern of the Au_0.5_/TiO_2_ NPs after the 1st, 3rd and 5th run were still demonstrated the main characteristic peaks of anatase- and rutile phase as described above. Besides, they exhibited the diffraction peaks of Au NPs at 2θ of 38.2, 44.4°, 64.6° and 77.6°, respectively corresponding to the face-centered cubic (FCC) structure of Au NPs at crystal plans of (111), (200), (220) and (311) (JCPDS No. 002-1095). More intense peak height was observed after a high repetitive run. By SEM–EDX analysis, the gold contents on the Au_0.5_/TiO_2_ after the 1st, 3rd and 5th run were around 2.18, 4.56 and 7.53 wt%, respectively. Despite the repetitive runs of gold recovery, an intensely uniform dispersion of Au NPs on the TiO_2_ surface was observed as illustrated in Fig. 16.

As proposed by Grieken et al.^6^, the deposited Au NPs can be separated from the TiO_2_ based material by the selective dissolution via (*i*) the aqua regia to leach metallic Au NPs, yielding the tetrachloroauric acid (HAuCl_4_) or (*ii*) hydrofluoric acid (HF) to leach the based catalyst, getting a brown gold power. To achieve the objective related to the gold recovery from industrial gold-cyanide plating wastewater, the selective dissolution of deposited Au NPs from the TiO_2_ was carried out using aqua regia as the extractant. By ICP analysis, the obtained solution contained gold ions of around 57, 140 and 224 mg/L, which were related to 2.19, 5.14 and 7.96 wt% Au on TiO_2_ after the 1st, 3rd and 5th run, respectively, consistent with those analyzed by SEM–EDX.

## Conclusion

A set of Au_*x*_/TiO_2_ NPs with different Au loadings was prepared via the photodeposition for photocatalytic gold recovery from industrial gold-cyanide plating wastewater. The deposited Au NPs in the investigated range of 0.1–1.0 wt% exhibited the insignificant effect on the A/R ratio, crystalline size of TiO_2_, surface area, but it affected importantly the size of deposited Au NPs as well as the light absorption capacity. Among all synthesized photocatalysts, the Au_0.5_/TiO_2_ NPs exhibited the best photocatalytic activity to recover gold from the industrial gold-cyanide plating wastewater. The addition of Na_2_S_2_O_3_ and C_1_-C_3_ alcohol as the hole scavengers promoted the photocatalytic gold recovery over the Au_0.5_/TiO_2_ NPs, while the H_2_O_2_ did not. Among all employed alcohols, the CH_3_OH exhibited the highest efficiency to promote the photocatalytic gold recovery. A complete recovery of gold ions can be achieved within 30 min at the photocatalyst dosage of 0.5 g/L, light intensity of 3.20 mW/cm^2^ in the presence of 20 vol% CH_3_OH as hole scavenger. The synthesized Au_0.5_/TiO_2_ NPs exhibited higher photocatalytic activity for gold recovery from the gold-cyanide containing solution than some photocatalysts in literature^6,20,52^. A very slight decrease of the photocatalytic gold recovery was observed after the 5th run, indicating its high reusability and high ability to accumulate the metallic Au NPs on its surface. The separation of deposited Au NPs from the used Au/TiO_2_ photocatalyst can be carried out by the selective dissolution using the chemical extractant. Besides, the repetitively used Au/TiO_2_ NPs are possibly used as the photocatalysts for other photocatalytic application such as H_2_ production^26,53–56^, but more characterizations and photocatalytic activity tests are required.

## Data Availability

All data generated or analyzed during this study are included in this published article.
